# Comparing extracellular vesicle enriched plasma proteomes between term and preterm neonates over the first days of life: post hoc analysis of a prospective observational study

**DOI:** 10.1136/bmjpo-2025-004393

**Published:** 2026-05-28

**Authors:** Daniel O’Reilly, Claire A Murphy, Luisa Weiss, Hayley Macleod, Ana Le Chevillier, Elaine Neary, John O’Loughlin, Afif EL-Khuffash, Barry Kevane, Fionnuala Ni Ainle, Naomi McCallion, Patricia B Maguire

**Affiliations:** 1 Paediatrics, Rotunda Hospital, Dublin, Ireland; 2 Conway Institute, University College Dublin, Dublin, Ireland; 3 Department of Neonatology, Chelsea and Westminster Hospital NHS Foundation Trust, London, UK; 4 Center for Interdisciplinary Cardiovascular sciences, Harvard Medical School, Boston, Massachusetts, USA; 5 Department of Neonatology, Liverpool Women’s Hospital, Liverpool, UK; 6 Department of Laboratory Medicine, Rotunda Hospital, Dublin, Ireland; 7 Department of Paediatrics, Royal College of Surgeons in Ireland, Dublin, Ireland; 8 Department of Neonatology, The Rotunda Hospital, Dublin, Ireland; 9 Department of Haematology, Mater Misericordiae University Hospital, Dublin, Ireland; 10 Haematology, Rotunda, Dublin, Ireland; 11 Neonatology, Rotunda Hospital, Dublin, Ireland; 12 Insight Research Ireland Centre for Data Analytics, University College Dublin, Dublin, Ireland

**Keywords:** Neonatology, Biochemistry, Molecular Biology

## Abstract

**Background:**

While cardiopulmonary transition in neonates is well described, molecular changes which accompany it are not. The Extracellular Vesicles in Early preterm neonates and Thrombin generation (EVENT) study sought to explore molecular transition in preterm birth. How this differs from term-born neonates is not well described.

**Methods:**

A post hoc evaluation of proteomic data generated from a subset of the prospective, single site observational EVENT study was performed comparing extracellular vesicle enriched plasma from term neonates (n=10) with preterm neonates (n=20) using a mass spectrometry, data dependent acquisition-based proteomic approach. Bioinformatics was performed using MaxQuant proteomics software and R statistical software.

**Results:**

Substantial differences were found between the preterm and term extracellular vesicle proteome, with proteins involved in extracellular matrix biology, immune function and embryogenesis all differentially expressed. Preterm neonates could be identified by their proteomic profile alone, reflecting their distinct physiology.

**Conclusions:**

These protein expression differences may underlie the pathogenesis of complications of prematurity, such as bronchopulmonary dysplasia, retinopathy of prematurity and intraventricular haemorrhage.

**WHAT IS ALREADY KNOWN ON THIS TOPIC**

**WHAT THIS STUDY ADDS**

- This study establishes, in a small cohort, that proteomic expression as analysed by mass spectrometry differs between term and preterm neonates during transition (days of life 1–3) in peripheral samples enriched for extracellular vesicles.

**HOW THIS STUDY MIGHT AFFECT RESEARCH, PRACTICE OR POLICY**

- Preterm molecular transition is a different process from term molecular transition. Molecular findings from a neonatal cohort at a given gestational age cannot necessarily be extrapolated based on day of life.

## Introduction

 Neonatal cardiopulmonary transition is a well-understood physiological phenomenon in healthy, term neonates. On taking their first breath, neonates expand their lungs, decreasing pulmonary vascular resistance. The umbilical cord is then cut, reducing the total capillary bed available for systemic blood flow and increasing systemic vascular resistance. This allows for the initial physiological closure of in-utero bypass circuits such as the ductus arteriosus, ductus venosus and foramen ovale, which then close anatomically and ideally remain closed for the remainder of life ex-utero.^1^

Molecular transition, or how circulating cells and blood components adapt to life outside the womb, is less well elucidated. Differences in coagulation profile have been noted in preterm infants compared with term infants in multiple studies, suggesting differences in proteomic content of blood.^2 3^ Neonatal coagulation has been demonstrated to be fundamentally different from coagulation in older children, with a greater dependency on high haematocrits and long von Willebrand factor polymers and reduced reliance on the traditional coagulation cascade and platelets. Longitudinal differences in protein expression in a preterm cohort have been demonstrated over multiple time points over the first weeks of life.^4^ Additionally, work focused on premature infants to date has suggested they have a prolonged and complicated molecular transition, with multiple studies showing differences over the first weeks of life.^5 6^ This molecular transition extends beyond the coagulation cascade, lymphocytes and platelets as described above, with differences also demonstrated between neonatal and adult monocytes and neutrophil function.^7^,^9^

Extracellular vesicles (EVs) are lipid-bound particles released from all cells, which contain proteins, lipids and microRNA.^10^ They are characterised by size, composition and cell of origin, with small EVs (50–150 nm) being predominantly involved in intercellular communication and large EVs (150 nm–1000 nm) involved in inflammation and coagulation.^10^ There has been a growing interest in the role of EVs in neonatology, particularly regarding neonatal coagulation, given the documented differences in neonatal clot formation.^11^

While the majority of the neonatal literature to date has used cord-derived blood as representative of day of life 1, there is growing evidence that there are key differences between cord blood-derived samples and peripheral blood-derived samples even early in neonatal life.^12^ Ontological differences have also been established in neonatal cellular development, with human fetal lymphocytes having a distinct expression profile from their adult equivalents.^13 14^ A recent study using cord blood-derived platelets from healthy full-term neonates and comparing them to those derived from adults showed substantial differences in the proteome and phosphoproteome from these two sources.^15^

Mass spectrometry offers a number of advantages for proteomic testing of neonatal blood with a requirement for reasonably small volumes of blood allowing for large numbers of proteins to be identified.^16^ There are also a number of limitations to the technique including the masking of low abundance, homologous protein sequences with post-translational modifications (eg, cytokines and chemokines) by high abundance, large proteins (eg, albumin).^17^ Enriching plasma sample for EVs helps reduce the level of albumin ‘masking’ of other biologically relevant proteins and offers insights into the physiological role of EVs. Importantly each EV retains a ‘halo’ of plasma so perfect isolation of EVs is thought to be biologically impracticable if not impossible.^18^ We therefore interpret findings in the context of an enriched, not fully purified, vesicle associated fraction.

Given the multiple comorbidities that occur in the context of preterm birth including chronic lung disease, retinopathy of prematurity and intraventricular haemorrhages, which are associated with immature vascular development, excessive fibrosis or inflammation, understanding the underlying physiology of neonatal transition is an important step for developing therapeutic strategies for this vulnerable group of patients.

This post-hoc analysis of data generated as part of the EVs in Early preterm Neonates and Thrombin generation (EVENT) study examined differences between preterm and term neonates using samples collected on day of life 1 and day of life 3 to establish how proteomic transition differs between preterm and term neonates.^19^ Our primary objective was to compare the EV enriched plasma proteome between preterm and term neonates during the first days of life. As a secondary objective, we examined whether proteomic changes between day of life 1 and day of life 3 differed within each cohort.

## Materials and methods

### Patient recruitment and blood collection

The EVENT Study was a prospective observational study carried out at a single tertiary maternity hospital (approx. 8500 deliveries/year) between September 2019 and December 2021. Recruitment was paused between March and June 2020 due to the outbreak of COVID-19. Ethical approval was obtained from the Rotunda Research Ethics Committee (REC-2019-012) and written informed parental consent was required for participation. Patients were not involved in the design or conduct of the study.

Preterm infants born between 24^+0^ weeks and 30^+6^ weeks gestation were eligible for inclusion in this study. Healthy full-term controls (37^+0^–42^+0^ weeks gestation) without major underlying anomalies were eligible for inclusion. Infants with a known antenatal brain haemorrhage or with a family history of coagulopathy were excluded.

Neonatal research samples were collected in the preterm group on day 1 (0–24 hours) and day 3 (48–72 hours). Neonatal blood samples were always collected at the same time as clinical samples. Samples were not collected after an infant had reached a corrected gestational age of 31^+0^ weeks, and irrespective of postnatal age, if an infant was not having a blood test performed for a clinical indication on that day or had received a blood product. Preterm EV proteomic datasets at days of life 1 and 3 have been reported previously as part of the EVENT study; the present analysis adds a term cohort processed and analysed using the same workflow to enable direct term-preterm comparison.^19^ Neonatal samples in the full-term group were collected at any time in the first 4 days of life (0–96 hours). For EV analysis in the term group, samples collected between 0–24 hours were considered day 1 samples, while those collected between 25–96 hours were day 3 samples. The research samples were collected in sodium citrate 3.2% of 0.5 mL or 1.3 mL size and gently inverted after collection. Blood samples were not taken from heparinised arterial lines and samples were hand-delivered directly to the laboratory.

### Preparation and storage of platelet poor plasma

All samples were processed as soon as possible after collection and within 4 hours. Each sample was manually inspected for evidence of clot formation and discarded if found. Platelet poor plasma (PPP) was generated by double centrifugation at 1187 xg for 6 min as per previously published recommendations for the removal of platelets from plasma.^20^ PPP was aliquoted into vials and stored between −70°C and −80°C.

### Sucrose cushion ultracentrifugation

Sucrose cushion ultracentrifugation was performed to create an EV enriched sample for proteomic analysis, a technique which has previously been demonstrated to yield a high purity of enriched EVs.^21^ A representative subset of PPP samples were taken from independent infants, that is, not paired samples, at two time points (preterm day 1 n=10, preterm day 3 n=10, term day 1 n=5, term day 3 n=5). 100 µL PPP was diluted with 850 µL cold Phosphate Buffered Saline (PBS). This was centrifuged at 8000 ×g for 10 mins at 4 °C. Supernatant was then transferred into an ultracentrifuge tube and a 50 µL solution of 30% sucrose/198 mM Tris-HCl was layered on the bottom. Samples were centrifuged at 51 000 rpm (equal to 120 000 ×g) at 4 °C for 6 hours (Beckman Coulter Ultramax Centrifuge with MLA 130 rotor, Beckman Coulter, California, USA). Supernatant was then carefully removed and sucrose cushion was transferred to a separate tube and resuspended in PBS. EVs were stored at −70 °C prior to mass spectrometry analysis.

### Immunoblotting

A total of 20 µg protein from each sample after ultracentrifugation was precipitated, resuspended in 2X RIPA lysis buffer (100 mM Tris pH 8.0, 300 mM NaCl, 2% Triton-X 100, 0.2% SDS, 1% sodium deoxycholate) containing protease inhibitors (Roche, Basel, Switzerland) and mixed with equal volumes of reducing sample buffer (125 mM TRIS HCl pH 6.7, 3% SDS, 7 mM dithiothreitol, 20% glycerol, 0.05% bromophenol blue). Proteins were separated on a 10% polyacrylamide gel before protein transfer onto a polyvinylidene fluoride membrane. Membranes were blocked in 5% skim milk for 1 hour at room temperature, washed 3×10 min with TBS-T (TBS+0.1% Tween-20) before incubation with primary antibodies at a dilution of 1:1000 overnight at 4 °C. CD63 and CD81 (Systems Biosciences, Palo Alto, California, USA), ApoA1 (R&D Systems, Abingdon, UK) and Albumin (Santa Cruz Biotechnology, Texas, USA) monoclonal antibodies were included to examine for EV markers and co-isolated plasma constituents. Following 3×10 min washes in TBS-T, membranes were incubated with fluorescently labelled secondary antibodies (LiCor Biosciences, Lincoln, Nebraska, USA) for 1 hour at room temperature. Proteins were detected using an Odyssey DLX Imaging system.

### Mass spectrometry

Mass spectrometry was conducted to evaluate the qualitative proteomic differences between EVs in preterm and term neonates.^22^ Mass spectrometry sample preparation was performed using the PreOmics iST HT 192x kit (P.O.00067) according to the manufacturer’s instructions. In brief, 100 ug of protein from each sample was lysed, reduced and alkylated for 10 min at 95 °C and 1000 rpm, transferred to a cartridge and subsequently double-digested with LysC and trypsin at 37 °C and 500 rpm for 1 hour. The digest was stopped, samples were washed twice with provided wash buffers and eluted using the provided elution buffer. Samples were evaporated at 45 °C and peptides resuspended in LC-load buffer to a final peptide concentration of 500 ng/µL. Further purification of the peptides was performed using Evotips (Evosep, Denmark). Each Evotip was activated by adding 25 µL of methanol, centrifuged at 700 ×g for 60 s with flow through collected in a 2 mL microcentrifuge tube. Next, 25 µL of buffer A (99.9% water/0.1% formic acid) was added and centrifuged as above. 10 µL sample was added to each Evotip and centrifuged as above. Finally, 120 µL of buffer A was added to wash and keep each tip wet. The tips were then stored at 4℃ prior to placing them on the Evosep One LC System. Samples were analysed using a Bruker timsTOF Pro mass spectrometer (Bruker, Billerica, Massachusetts, USA) connected to an Evosep One LC system and separated with an increasing acetonitrile gradient over 40 min at a flow rate of 250 nL/min at 45 °C. The mass spectrometer was operated in positive ion mode with a capillary voltage of 1500 V, dry gas flow of 3 L/min and a dry temperature of 180 °C. All data were acquired with the instrument operating in trapped ion mobility spectrometry mode. Trapped ions were selected for tandem mass spectrometry (MS/MS) using parallel accumulation serial fragmentation (PASEF). A scan range of (100–1700 m/z) was performed at a rate of 10 PASEF MS/MS frames to 1 MS scan with a cycle time of 1.16 s.

Tandem mass spectra were searched against a human FASTA (Uniprot, June 2021) using the freely available MaxQuant proteomics software (V.1.6.17.0) as before.^22^ MaxQuant analysis included an initial search with a precursor mass tolerance of 20 ppm the results of which were used for mass recalibration. In the main Andromeda search precursor mass and fragment mass had an initial mass tolerance of 6 ppm and 20 ppm, respectively. The search included fixed modification of carbamidomethyl cysteine. Minimal peptide length was set to seven amino acids, and a maximum of two miscleavages was allowed. The false discovery rate (FDR) was set to 0.01 for peptide and protein identifications. MaxQuant assigns label-free quantitative values to each identified protein.

### Statistical analysis

Statistical analysis on EV proteomic contents was performed as follows and as described previously.^22^ Initial statistical analysis including significance testing was performed using Perseus software (Max Planck Institute of Biochemistry, Bayern, Germany).^23^ Only proteins identified in at least 50% of the replicate runs in at least one time point or population were included. Differences in protein expression were determined using an unpaired t test with an FDR of 5% and a minimal fold change of 0.1 within Perseus software. Dataset was then analysed using R statistical software (V.4.2.2, The R Foundation for Statistical Computing, Vienna, Austria). Proteomic data set were imputed using quantile regression imputation for left censored data and principal component analysis was performed. A volcano plot was generated using ‘EnhancedVolcano’. The ‘Pheatmap’ package was used to generate heatmap, clustering was performed using Euclidean distance for proteins and Ward’s criterion (WardD2) for patients. Gene set enrichment analysis (GSEA) was performed using the package ‘clusterprofiler’ comparing against the Gene Ontology database.

## Results

### Patient demographics

Preterm samples on day of life 1 (n=10) and day of life 3 (n=10) were analysed as previously described.^19^ The median corrected gestational age of neonates in the day of life 1 cohort was 28.1 weeks (IQR 27–29.1 weeks) and in the day of life 3 cohort was 29.3 weeks (IQR 27.9–30 weeks). Median birth weight in the day of life 1 cohort was 1047.3 g (IQR 760–1210 g), and in the day of life 3 cohort, this was 1302.5 g (IQR 890–1470 g). The term cohort on both days of life 1 and 3 had a median gestational age of 39.29 (IQR 38.9–39.9 weeks) and birth weight of 3445 g (IQR 3200–3830) as described previously.^19^

### Protein content of EV-enriched platelet-poor plasma differs between term and preterm neonates

**Figure 1:**
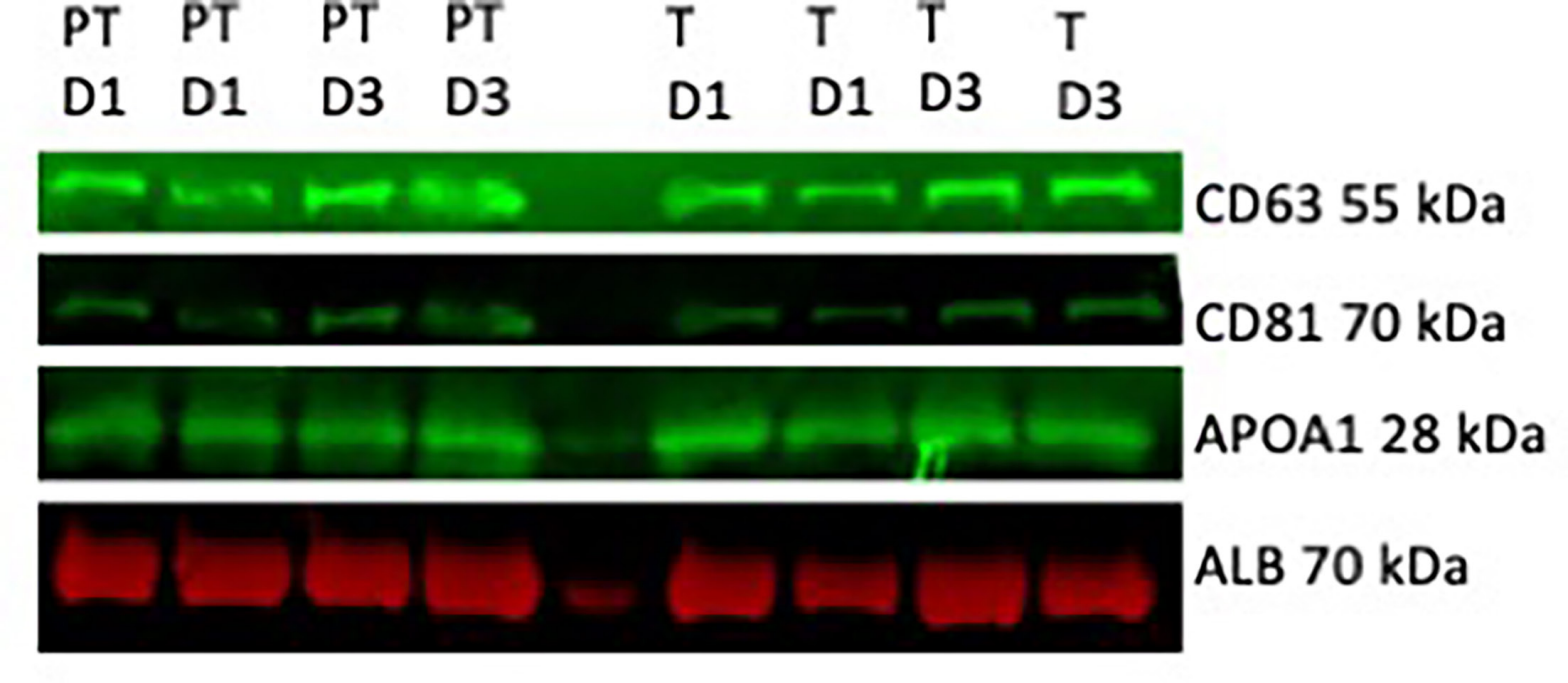
Western blot demonstrating enrichment for extracellular vesicle-specific markers (CD63 and CD81) and plasma markers (ApoA1 and albumin) in preterm samples (PT) and term samples (**T**) on day 1 (**D1**) and day 3 (**D3**) of life.

**Figure 2:**
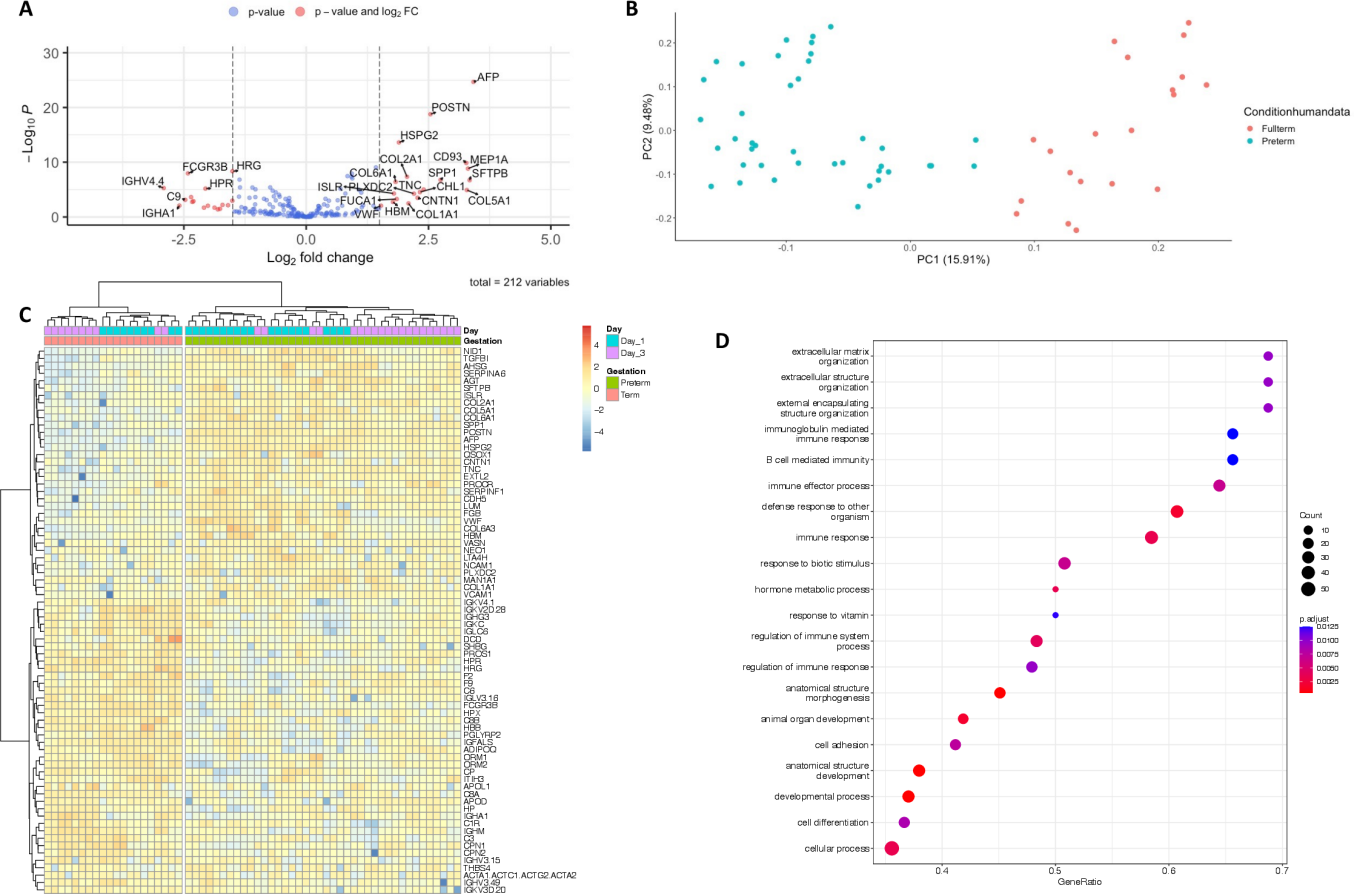
(A) Volcano plot demonstrating relative changes in expression between preterm and term infants in proteomic expression, proteins upregulated in preterm infants are represented as having a positive log fold change (FC) and proteins upregulated in term infants are represented as having a negative log FC. (B) Principal component analysis (PCA) demonstrating separation of term (red) and preterm (blue) neonates. (C) Heatmap demonstrating clustering of term (red) and preterm (green) neonates, note limited clustering based on day of life 1 (blue) and day of life 3 (purple). (D) Dotplot demonstrating enriched biological pathways by Gene Set Enrichment Analysis (GSEA) against Gene Ontology terms comparing preterm neonates and term neonates over the first 3 days of life, no differential analysis was possible between day 1 and day 3.

**Figure 3:**
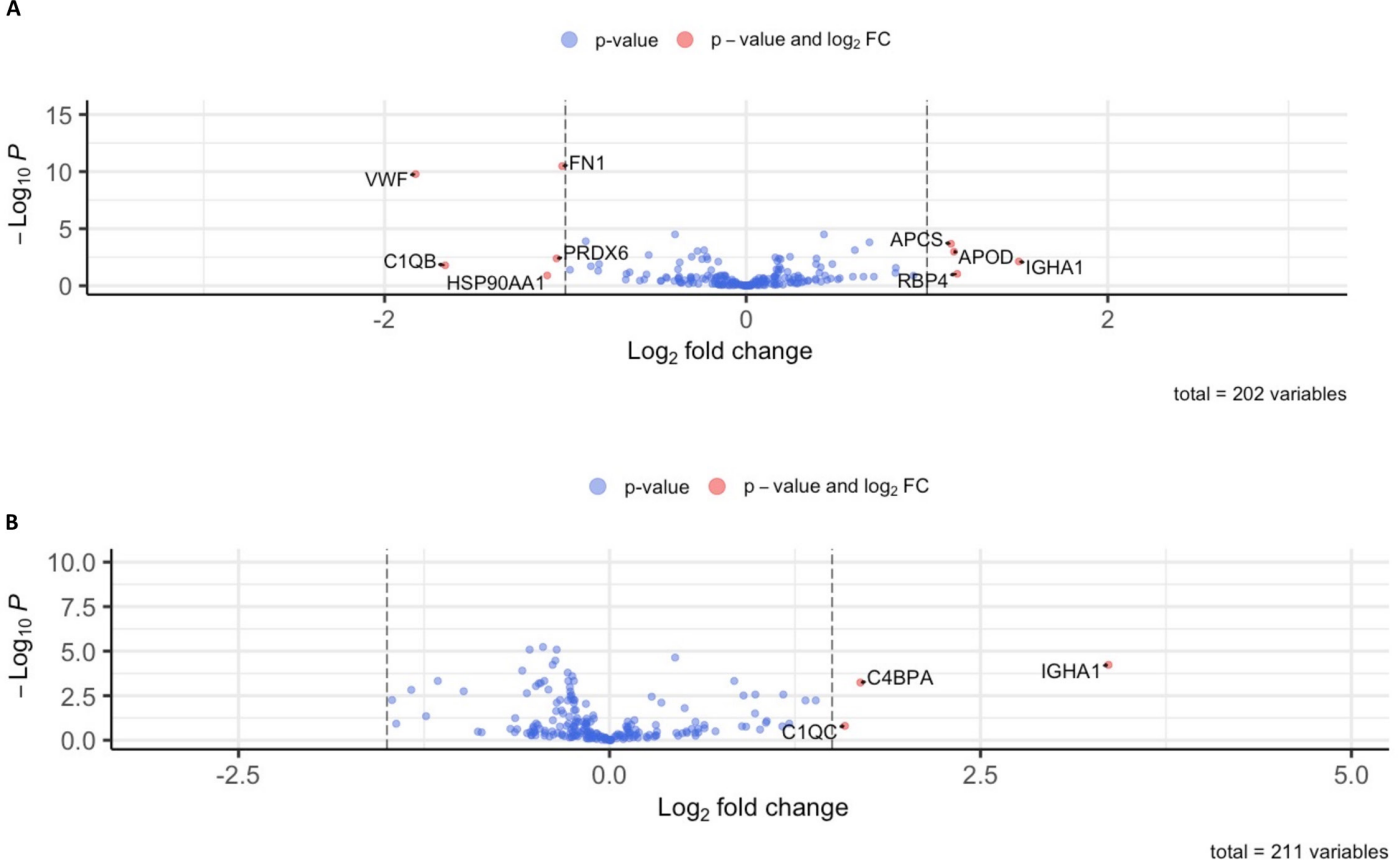
Volcano plots demonstrating limited proteomic differences between (A) preterm infants on day of life 1 and day of life 3 and (B) term infants on day of life 1 and day of life 3. Note no gene set enrichment analysis is possible as no pathway was enriched in a statistically significant (p<0.05) manner in either term or preterm neonates over the first 3 days of life. Proteins which are upregulated on day 3 are depicted as positive fold change while proteins upregulated on day 1 are shown as negative fold change. (A) previously published with different thresholds in Murphy *et al.*^19^

EVs were enriched from PPP, as confirmed by immunoblotting for EV markers (CD63 and CD81, figure 1), and subjected to mass spectrometry. Biophysical properties of EVs between these two groups have been described in a previous publication and are summarised in online supplemental table S2.^19^ A total of 212 proteins were identified with 18 proteins being significantly upregulated in our preterm cohort relative to our term cohort and 19 significantly downregulated in our preterm cohort relative to our term cohort (fold change of +/−1.5, adjusted p value of >0.05, figure 2, online supplemental table S1). Of the included proteins, two were only found in a preterm population (COL15A1, MEP1A) and one protein was only identified in term neonates (CA1). The remaining 209 proteins were identified across both groups. Multivariate analysis using both principal component analysis and hierarchical clustering demonstrated that proteomic data clustered according to preterm birth and term birth rather than day of life (figure 2). This was further confirmed by GSEA analysis comparing day 1 and day 3 of life in both preterm and term neonates, which showed no statistically enriched pathways between day 1 and day 3 in preterm and term neonates. A small number of statistically and fold change significant differences between proteins on day of life 1 and day of life 3 in both preterm and term neonates are shown in figure 3.

### Distinct pathways differ between preterm and term neonates on gene set expression analysis

To establish what likely biological pathways were different between term and preterm neonates, we performed GSEA on the proteomic data. A number of pathways were upregulated in a statistically significant way (p<0.05), and these are outlined as a dotplot in figure 2. Notably, pathways involving extracellular matrix proteins, immune function and embryogenesis were significantly different between the two cohorts.

## Discussion

This paper outlines the distinct proteomic differences that exist between preterm and term neonates in an EV-enriched plasma sample. Previous work has noted differences in immune proteins and cells between term and preterm neonates, which showed convergence of immune profiles over time. Other work has documented the significant changes in plasma protein profile over the first weeks of life in preterm neonates, suggesting both a prolonged and complicated transition to extrauterine life.^6^ Within the EVENT study, we have previously demonstrated that there are distinct EV populations between day 1 and day 3 of preterm neonates, which are not recapitulated in term neonates, suggesting molecular differences in transition.^19^ Proteomic changes over this time period have previously been noted to be limited, and this study demonstrated similarly minor changes over the first days of life.^1^ Age-dependent changes have been demonstrated separately in term neonates versus older children and adults, and between preterm neonates and adults, predominantly using either whole blood or plasma.^4 24 25^ This study builds on this existing data by demonstrating, for the first time, distinct differences in relative expression between transitioning preterm and term newborns of the same chronological age using an EV-enriched, peripherally derived plasma sample.

The differences observed between preterm and term neonates offer valuable insights into the distinct physiological transitions in each group. Upregulation of extracellular matrix proteins is interesting from an embryogenesis and fibrosis standpoint. For example, Tenascin C (TNC) is associated with embryogenesis and adult cardiac dysfunction, which is relevant given the growing interest in the long-term cardiac consequences of preterm birth.^26 27^ TNC is also implicated in the pathophysiology of bronchopulmonary dysplasia (BPD) with a hyperoxic murine model of BPD with pups raised in a hyperoxic environment having a higher concentration of TNC versus those raised in a normoxic environment.^28^ Collagens are intrinsically related to the development of BPD with high levels of collagen I, components of which are upregulated in this analysis, being specifically upregulated in BPD and normal branching architecture being preserved in the absence of collagen I.^29 30^ Indeed, one current treatment aimed at reducing the occurrence of BPD in this population, postnatal dexamethasone, has been demonstrated to reduce collagen synthesis.^31^

Several proteins upregulated in the preterm cohort (cell adhesion molecule L1 like/CHL1, Contactin 1/CNTN1) are associated with neuronal migration and development. In this healthy cohort, these may reflect the rapid changes and development of the preterm neonatal brain as deletion of CHL1 is associated with language and cognitive learning difficulties in children.^32^ *Cntn1* gene expression has been found to be downregulated in umbilical cord blood from neonates exposed to fetal inflammatory response syndrome (FIRS).^33^ Exposure to FIRS has been associated with poorer neurodevelopmental prognosis.^34^ Thus, upregulation of these proteins may represent a neuroprotective response in preterm birth.

Our findings complement other similar proteomic studies emphasising many of the factors involved in preterm transition. Relative deficiency of immune proteins such as immunoglobulins and complement have been documented in other studies while the overexpression of alpha-fetoprotein and von Willebrand factor is well established in the literature.^35^,^38^ This verification of known factors associated with neonatal proteomic profiles is reassuring that the observed changes in other more novel pathways such as extracellular matrix proteins are likely to represent true biological change. Importantly, this is the first study we could identify in the literature to date to examine proteomic differences by mass spectrometry between a healthy preterm and term cohort using a peripherally derived blood sample as opposed to cord blood sampling (table 1). Other studies have established the lack of equivalence between cord blood and peripheral blood samples even on day of life 1 from neonates using other methods.^12^

**Table 1:** Comparison with a sample of previous published work examining preterm neonatal proteomics

Authors	Proteomic methods used	Sample source	Sample population	Comparison population
Bjelosevic *et al*^25^	*SWATH MS*	*Peripheral blood*	*Term neonates >37 weeks*	*Healthy older children and adults*
Suski *et al*^39^	iTRAQ	Cord blood	*Preterm neonates <30 weeks*	*Full term neonates*
Olin *et al*^12^	*PEA*	*Cord and peripheral blood*	*Preterm neonates <30 weeks*	*Full term neonates*
Smit *et al*^4^	*DIA-NN*	*Cord and peripheral blood*	*Preterm neonates <30 weeks who were SGA or AGA*	*Healthy adults*
Thom *et al*^15^	*DIA*	*Cord blood (platelet isolates*)	*Term neonates*	*Healthy adults*

AGA, Appropriate for gestational age; DIA, Data Independent Acquisition; DIA-NN, Data Independent Acquistion with Neural Network; iTRAQ, isobaric Tags for Relative and Absolute Quatitation; PEA, Proximity Extension Analysis; SGA, Small for Gestational Age; SWATH MS, Sequential Window Acquisition of all Theoretical fragment ion spectra mass spectrometry.

There are a number of limitations to this study. This cohort was recruited primarily to examine physiological transition between term and preterm neonates and therefore represents a reasonably ‘healthy’ preterm cohort. While this reduces confounding due to the transfusion of blood products or immediate complications of prematurity, it limits our ability to identify possible markers of disease in this cohort. Due to the limited plasma volumes obtained from each neonate, full EV isolation was not feasible; instead, an enriched sample was used, which also contained plasma proteins and lipid particles (figure 1). Similarly, due to limitations with both sample availability and total numbers required for a robust statistical analysis, a paired longitudinal analysis was not feasible.

This work develops on previous proteomic profiling of preterm neonates by establishing that preterm transition is biologically distinct from term neonatal transition. Differences in immune function, extracellular matrix proteins and embryogenesis predominate over the first days of life and may explain comorbidities related to preterm birth.

## Supplementary material

10.1136/bmjpo-2025-004393 online supplemental file 1

10.1136/bmjpo-2025-004393 online supplemental file 2

## Data Availability

Data are available in a public, open access repository. Data are available upon reasonable request.
