# Supplementary material for: Comparing extracellular vesicle enriched plasma proteomes between term and preterm neonates over the first days of life: post hoc analysis of a prospective observational study
Source: BMJ Paediatr Open. 2026 May 28;10(1):e004393. doi: 10.1136/bmjpo-2025-004393 (PMC13223661; doi:10.1136/bmjpo-2025-004393)
Supplement: online supplemental file 2 [file bmjpo-10-1-s002.docx]

Supplementary Table 2

|  | Preterm | | Term | |
| --- | --- | --- | --- | --- |
|  | Day 1  Median (IQR) | Day 3  Median (IQR) | Day 1  Median (IQR) | Day 3  Median (IQR) |
| Number of samples | 69 | 28 | 5 | 12 |
| Concentration of small EVs, particles/ml | 1.5 x 10^10^  (1.16 x 10^10^ –  2.35 x 10^10^) | 1.07 x 10^11^  (6.74 x 10^10^ –  1.48 x 10^11^) | 5.42 x 10^10^  ( 4.82 x 10^10^-  5.98 x 10^10^) | 6.51 x 10^10^  (5.26 x 10^10^ –  7.78 x 10^10^) |
|  |  |  |  |  |
| Number of samples | 23 | 11 | 5 | 11 |
| Concentration of large EVs, particles/µl | 628.7  (383.9 – 1390.7) | 20674.3  (18414.7 – 35689.5) | 703.6  (528.6 –  890.9) | 1590.7  (1077.9 –  2522) |

Supplementary Table 2: Concentration of small extracellular vesicles (<200 nm) measured by nanoparticle tracking analysis and large extracellular vesicles measured by flow cytometry in preterm and term infants in the EVENT cohort

*EVs- extracellular vesicles*
