# Supplementary material for: Comparing extracellular vesicle enriched plasma proteomes between term and preterm neonates over the first days of life: post hoc analysis of a prospective observational study
Source: BMJ Paediatr Open. 2026 May 28;10(1):e004393. doi: 10.1136/bmjpo-2025-004393 (PMC13223661; doi:10.1136/bmjpo-2025-004393)
Supplement: Supplementary file 3 [file bmjpo-2025-004393.reviewer_comments.pdf]

PEER REVIEW HISTORY

BMJ Paediatrics Open publishes all reviews undertaken for accepted manuscripts. Reviewers are asked to complete a checklist review form and are provided with free text boxes to elaborate on their assessment. These free text comments are reproduced below.

ARTICLE DETAILS

**TITLE (PROVISIONAL)**	Comparing Extracellular Vesicle enriched plasma proteomes between term and preterm neonates over the first days of life: posthoc analysis of a prospective observational study
**AUTHORS**	O'Reilly, Daniel<br>Murphy, Claire<br>Weiss, Luisa<br>Macleod, Hayley<br>Le Chevillier, Ana<br>Neary, Elaine<br>O'loughlin, John<br>EL-Khuffash, Afif<br>Kevane, Barry<br>Ni Ainle, Fionnuala<br>McCallion, Naomi<br>Maguire, Patricia B

VERSION 1 – REVIEW

**REVIEWER NAME**	Davenport, Patricia
**REVIEWER AFFILIATION**	
**REVIEWER CONFLICT OF INTEREST**	No Conflict of Interest
**AI DISCLOSURE**	No, I have not used AI
**DATE REVIEW RETURNED**	13-Jan-2026

**GENERAL COMMENTS**	In this post hoc analysis of proteomic data from the EVENT study, the authors investigate differences in the protein content of EV enriched plasma between preterm and term infants as they undergo the significant transition between day of life 1 and 3. This study is very interesting but fails to answer the primary question. I think it can be greatly enhanced by the provision of additional background information and a more structured/detailed analysis and discussion of the interesting data generated.<br><br>Major Comments:<br>1. The introduction requires reorganization and significant additions to introduce the concepts required to understand the manuscript:<br><br>i. First, the authors need to describe the concept of 'molecular transition' and describe what EVs are and why EV enriched plasma was used in more detail for a reader unfamiliar with both concepts. Reference 11 (The EVENT study by this same group) nicely puts this all into context for the reader, but the authors should not assume readers will seek out this publication.<br>ii. Second, reference 2 would likely be better positioned at the end of the paragraph supporting the ontological differences that have been described between neonatal and adult cells (along with references 9 and 10). In addition to platelets and lymphocytes, groups have also

	performed comparisons between neonatal and adult monocytes and neutrophils (and likely others) that could similarly be supportive of the statement the authors are trying to make. Reference 3 could stand alone in support of the rapid transition seen in preterm neonates, but please provide more information about the study as it is not clear that it is a comparison of serum proteins over preterm transition.<br><br>2. Are the preterm samples on day 1 and 3 the same samples previously analyzed and published in reference 11? If so, this needs to be made clear in the Methods and in the Results by specifying that this is a comparison of the term data to previously published preterm data.<br><br>3. In the methods, the authors state that these are “independent” samples, which I think means that the day 1 and day 3 samples come from different infants in both cohorts. This should be stated with more transparency and mentioned in the limitations section as it is critical to the interpretation of the data.<br><br>4. Despite the focus of the manuscript being on comparing the ex-utero transition from days 1 and 3 between term and preterm infants, the Results section barely mentions the data regarding this analysis, other than to say that proteins clustered more by preterm vs term birth than by day – but that does not answer the question of if preterm and term infants transition from day 1 to 3 differently. I assume the author’s hypothesis is that they do transition differently, and so you would not expect the combined proteomic data from both groups to separate similarly based on day of sample (that would suggest they transition similarly), unless the authors meant that there were not 4 separate clusters. Thus, the Results section needs to be significantly enhanced first, by first providing data on how the proteome changes over the day 1-3 transition in preterm (which I think was already published in reference 11) and term infants (which is what this manuscript is adding to the literature) separately- which should include separate volcano plots comparing day 1 and 3 in each population. GSEA analysis could be conducted during this section as well in this section, to compare what pathways are being altered in each cohort during this transition. The current Results section (which I think is comparing the entire EV proteome between preterm and term infants regardless of day?) is interesting and should be retained but requires clarification as to what is being compared but, if my interpretation is correct, is not the primary question being asked in this manuscript, and thus should be secondary.<br><br>5. Does the GSEA analysis in Figure 2D depict the preterm or term proteome? A GSEA analysis of both should be provided to facilitate comparison.<br><br>6. In the Results, the Discussion, the volcano plots and the supplemental table 1- the authors need to provide directionality to all their findings and discussions. Which way is the protein of interest enriched – is it higher in term vs. preterm? And at what day or is it a combination? What is being compared in supplemental Table 1? It is otherwise impossible for a reader to interpret your findings.<br><br>7. Similar to the Results section, in the Discussion the authors should separately and clearly describe first, their interpretation of how the two groups differ over the day 1-3 transition (the primary

	question of the paper). This should include an interpretation of specific proteins or pathways (if a GSEA analysis is done) found to be different between the two groups. Next, the authors can move into an interpretation of proteins found to be enriched in the overall proteome between preterm and term patients (which I think is what it currently is?). This should also include a robust description of proteins found to be up or down regulated in both populations. In its current state, the authors only highlight very few proteins that are (I think) enriched in the preterm population and are missing the opportunity to highlight the massive number of immune/inflammatory proteins that are enriched in term compared to preterm patients. This could have its own section in the discussion since risk of major infection and the ability to mount an antibody response are 2 very important differences between preterm and term infants.<br><br>Minor Comments:<br>1. The title is bulky and does not describe that this is a comparison of the protein content of EV enriched plasma between term and preterm infants.<br>2. Please provide a reference for the second sentence in the Discussion.<br>3. In describing reference 20 in the Discussion, please specify that there is an increase in TNC in pups exposed to hyperoxia compared to those raised in normoxia.

**REVIEWER NAME**	Ransom, Meaghan
**REVIEWER AFFILIATION**	
**REVIEWER CONFLICT OF INTEREST**	No Conflict of Interest
**AI DISCLOSURE**	No, I have not used AI
**DATE REVIEW RETURNED**	15-Feb-2026

**GENERAL COMMENTS**	Overall I felt this manuscript needed to be proofed for grammatical errors throughout before submitting. Minor examples like sometimes EV is written and other times it is spelled out, words missing between commas to merge sentences, etc. The abstract is not very clear and concise and I would suggest making substantial changes to the abstract. Specifically, the background sentences do not flow well. In the methods section it would help the reader to state these were a subset of patients. I would not include the comment about power in the conclusion section and discuss this as a limitation of the study later in the manuscript.<br>The main data figure is the highlight of the manuscript. Can you make the datapoints in B bigger and also the legend text bigger for B? For C, I would move the legend closer to the data.

VERSION 1 – AUTHOR RESPONSE

Reviewer: 1
Patricia Davenport

Comments to the Author

In this post hoc analysis of proteomic data from the EVENT study, the authors investigate differences in the protein content of EV enriched plasma between preterm and term infants as they undergo the significant transition between day of life 1 and 3. This study is very interesting but fails to answer the primary question. I think it can be greatly enhanced by the provision of additional background

information and a more structured/detailed analysis and discussion of the interesting data generated. We thank Reviewer 1 for a careful and constructive evaluation. We agree that the original framing did not make the primary objective sufficiently explicit, which could reasonably lead to the impression that our manuscript does not answer its intended question. We have therefore rewritten the final paragraph of the Introduction and the opening of the Results and Discussion to state clearly that the primary analysis is a term versus preterm comparison of the EV-enriched plasma proteome during transition in the early postnatal period, with day-of-life comparisons within each cohort treated as secondary. To make our manuscript's logic easier to follow, we also restructured the Results to mirror this and revised the Discussion accordingly. These changes are designed to answer the primary question directly and transparently, in line with Reviewer 1's excellent recommendation.

Major Comments:
1. The introduction requires reorganization and significant additions to introduce the concepts required to understand the manuscript:

i. First, the authors need to describe the concept of 'molecular transition' and describe what EVs are and why EV enriched plasma was used in more detail for a reader unfamiliar with both concepts. Reference 11 (The EVENT study by this same group) nicely puts this all into context for the reader, but the authors should not assume readers will seek out this publication.
Many thanks for this comment, we agree it will be important to supply the reader new to this literature with more context. We have therefore expanded the introduction considerably to (i) define "molecular transition" in the context of neonatal adaptation to extrauterine life, (ii) provide a concise operational definition of extracellular vesicles (EVs) and appropriate nomenclature, and (iii) justify the use of an EV-enriched plasma preparation (including a clear statement of what "enriched" does and does not imply regarding purity).

Manuscript changes p4-6

ii. Second, reference 2 would likely be better positioned at the end of the paragraph supporting the ontological differences that have been described between neonatal and adult cells (along with references 9 and 10). In addition to platelets and lymphocytes, groups have also performed comparisons between neonatal and adult monocytes and neutrophils (and likely others) that could similarly be supportive of the statement the authors are trying to make. Reference 3 could stand alone in support of the rapid transition seen in preterm neonates, but please provide more information about the study as it is not clear that it is a comparison of serum proteins over preterm transition.

Thank you very much for your thoughtful and constructive comments. We fully recognise the importance of comprehensively outlining the differences between adult and neonatal monocytes and neutrophils. However, given the scope and focus of this manuscript, it is challenging to include a comprehensive overview within the constraints of a short introduction alongside the existing content. That said, in response to your helpful feedback, we have strengthened this section by explicitly acknowledging the breadth of the literature in this area and by incorporating a selection of additional key references. We hope this provides an appropriate balance between context and focus. Specifically, we have added the following text on page 04 of the manuscript: "This extends beyond the coagulation cascade, lymphocytes and platelets with differences also demonstrated between neonatal and adult monocytes and neutrophil function[8-10]"

2. Are the preterm samples on day 1 and 3 the same samples previously analyzed and published in reference 11? If so, this needs to be made clear in the Methods and in the Results by specifying that this is a comparison of the term data to previously published preterm data.
Thank you. We agree this must be explicit. We now clearly state in the Methods and again in the

Results demographics section which preterm samples/timepoints were previously analysed as part of the EVENT study and which analyses/data are novel in the present manuscript. We also clarified this in the figure/table captions where relevant to avoid any ambiguity about what is being newly compared.

Manuscript changes
p.7 “Preterm EV proteomic datasets at day of life 1 and 3 have been reported previously as part of the EVENT study; the present analysis adds a term cohort processed and analysed using the same workflow to enable direct term–preterm comparison”

Legend figure 3: “3A previously published with different thresholds in Murphy et al.2025”

3. In the methods, the authors state that these are “independent” samples, which I think means that the day 1 and day 3 samples come from different infants in both cohorts. This should be stated with more transparency and mentioned in the limitations section as it is critical to the interpretation of the data.
We agree, and we have revised the Methods to state explicitly that the day of life 1 and day of life 3 samples are from different infants (i.e., this is not a paired longitudinal analysis). We also added a brief statement in the limitations section explaining that limited sample availability and the requirements for robust longitudinal inference precluded a paired within-infant proteomic trajectory analysis in this work. This clarification is important for correct interpretation of the day-of-life comparisons.

Changes:
p.8: “i.e not paired samples”
p.14: “Similarly due to limitations with both sample availability and total numbers required for a robust statistical analysis, a paired longitudinal analysis was not feasible.”

4. Despite the focus of the manuscript being on comparing the ex-utero transition from days 1 and 3 between term and preterm infants, the Results section barely mentions the data regarding this analysis, other than to say that proteins clustered more by preterm vs term birth than by day – but that does not answer the question of if preterm and term infants transition from day 1 to 3 differently. I assume the author’s hypothesis is that they do transition differently, and so you would not expect the combined proteomic data from both groups to separate similarly based on day of sample (that would suggest they transition similarly), unless the authors meant that there were not 4 separate clusters. Thus, the Results section needs to be significantly enhanced first, by first providing data on how the proteome changes over the day 1-3 transition in preterm (which I think was already published in reference 11) and term infants (which is what this manuscript is adding to the literature) separately- which should include separate volcano plots comparing day 1 and 3 in each population. GSEA analysis could be conducted during this section as well in this section, to compare what pathways are being altered in each cohort during this transition. The current Results section (which I think is comparing the entire EV proteome between preterm and term infants regardless of day?) is interesting and should be retained but requires clarification as to what is being compared but, if my interpretation is correct, is not the primary question being asked in this manuscript, and thus should be secondary.

This comment from reviewer 1 was particularly helpful as it highlighted where our structure allowed two differential readings. We have now addressed this in two ways. First, we revised the Introduction and Results headers to state unambiguously that the primary comparison is term versus preterm EV-enriched plasma proteomes during early postnatal life. Second, to address the reviewer’s request

directly, we added the within-cohort day of life 1 versus day of life 3 comparisons as a secondary analysis (new Figure 3), using the same statistical thresholds for consistency. These within-cohort comparisons show that day-of-life proteomic changes over this short window are modest in both cohorts, and, consistent with those limited changes, GSEA for day-of-life comparisons does not meet significance thresholds at the chosen FDR.

We made these specific changes to the manuscript on page 6 “Our primary objective was to compare the EV enriched plasma proteome between preterm and term neonates during the first days of life. As a secondary objective, we examined whether proteomic changes between day of life 1 and day of life 3 differed within each cohort.”
Page 12 “Within the EVENT study, we have previously demonstrated that there are distinct extracellular vesicle populations between day 1 and day 3 of preterm neonates which are not recapitulated in term neonates, suggesting molecular differences in transition”
Addition of Figure 3: : “Volcano plots demonstrating limited proteomic differences between A) preterm infants on day of life 1 and day of life 3 and B) term infants on day of life 1 and day of life 3. Note no gene set enrichment analysis is possible as no pathway was enriched in a statistically significant ($p < 0.05$) manner in either term or preterm neonates over the first 3 days of life. Proteins which are upregulated on day 3 are depicted as positive fold change while proteins upregulated on day 1 are shown as negative fold change. 3A previously published with different thresholds in Murphy et al.2025”

5. Does the GSEA analysis in Figure 2D depict the preterm or term proteome? A GSEA analysis of both should be provided to facilitate comparison.

We agree the GSEA analysis must be unambiguous. We clarified in the Results text and figure caption that the displayed enrichment reflects the ranked differential expression between preterm versus term (i.e., a between-cohort comparison), and we labelled directionality in the plot/legend accordingly. In addition, we now explicitly state that within-cohort day-of-life comparisons do not yield significant enrichment at the chosen FDR, and we therefore do not present a dot plot for those null comparisons.

Specific manuscript changes: Legend to figure 2 “Figure 2A-D: A) Volcano plot demonstrating relative changes in expression between preterm and term infants in proteomic expression, proteins upregulated in preterm infants are represented as having a positive log fold change and proteins upregulated in term infants are represented as having a negative log fold change B) Principal component analysis (PCA) demonstrating separation of term (red) and preterm (blue) neonates. C) Heatmap demonstrating clustering of term (red) and preterm (green) neonates, note limited clustering based on day of life 1 (blue) and day of life 3 (purple).D) Dotplot demonstrating enriched biological pathways by Gene Set Enrichment Analysis (GSEA) against Gene Ontogeny (GO) terms comparing preterm neonates and term neonates over the first 3 days of life, no differential analysis was possible between day 1 and day 3”

6. In the Results, the Discussion, the volcano plots and the supplemental table 1- the authors need to provide directionality to all their findings and discussions. Which way is the protein of interest enriched – is it higher in term vs. preterm? And at what day or is it a combination? What is being compared in supplemental Table 1? It is otherwise impossible for a reader to interpret your findings.
Figure clarified in supplemental table and diagrams.

We agree and have corrected this throughout. Specifically, we revised the Results narrative and all relevant figure legends to state directionality explicitly (e.g., “higher in preterm relative to term” or “higher in term relative to preterm”), and we ensured the Tables include clear column headers indicating comparison direction and effect size. This enables readers to interpret findings without inference from axes alone and avoids ambiguity about whether “upregulated” refers to preterm or term.

Specific manuscript changes: Legend to figure 2 “Figure 2A-D: A) Volcano plot demonstrating relative changes in expression between preterm and term infants in proteomic expression, proteins upregulated in preterm infants are represented as having a positive log fold change and proteins upregulated in term infants are represented as having a negative log fold change B) Principal component analysis (PCA) demonstrating separation of term (red) and preterm (blue) neonates. C) Heatmap demonstrating clustering of term (red) and preterm (green) neonates, note limited clustering based on day of life 1 (blue) and day of life 3 (purple).D) Dotplot demonstrating enriched biological pathways by Gene Set Enrichment Analysis (GSEA) against Gene Ontogeny (GO) terms comparing preterm neonates and term neonates over the first 3 days of life, no differential analysis was possible between day 1 and day 3”

Supplemental data legend:” All 212 proteins identified alongside unadjusted p-values, adjusted p-values by the Benjamini-Hochberg method, T statistic, Average expression and Log fold change of Label free quantification units (LFQs) positive fold change indicates relative overexpression in preterm neonates while negative fold change indicates relative overexpression in term neonates

7. Similar to the Results section, in the Discussion the authors should separately and clearly describe first, their interpretation of how the two groups differ over the day 1-3 transition (the primary question of the paper). This should include an interpretation of specific proteins or pathways (if a GSEA analysis is done) found to be different between the two groups. Next, the authors can move into an interpretation of proteins found to be enriched in the overall proteome between preterm and term patients (which I think is what it currently is?). This should also include a robust description of proteins found to be up or down regulated in both populations. In its current state, the authors only highlight very few proteins that are (I think) enriched in the preterm population and are missing the opportunity to highlight the massive number of immune/inflammatory proteins that are enriched in term compared to preterm patients. This could have its own section in the discussion since risk of major infection and the ability to mount an antibody response are 2 very important differences between preterm and term infants.

The primary question of this paper is the difference in term versus preterm proteomes over the first 3 days of life, not the difference between day 1 and day 3 in either group for reasons discussed above. Volcano plots as described are explanations of relative expression, directionality has been explicitly stated as requested. Our data did not support a “massive number of immune/inflammatory” proteins being upregulated as we only noted relative abundance of immunoglobulins and complement (note cytokines/chemokines are poorly described by mass spectrometry based techniques for a number of reasons well beyond the scope of this paper but including sequence homology, relatively low abundance and post translational modifications). Hopefully the additional comments on directionality and on supplemental table make this clear

Minor Comments:
1. The title is bulky and does not describe that this is a comparison of the protein content of EV enriched plasma between term and preterm infants.
Agreed. We shortened the title to state the comparison and sample type directly, and removed excess qualifiers that did not add information while adhering to journal formatting. It now reads as follows

“Comparing Extracellular Vesicle enriched plasma proteomes between term and preterm neonates over the first days of life:A posthoc analysis of a prospective observational study”
2. Please provide a reference for the second sentence in the Discussion.
We added an appropriate citation to support the statement. It is now reference 7 in the revised manuscript.

3. In describing reference 20 in the Discussion, please specify that there is an increase in TNC in pups exposed to hyperoxia compared to those raised in normoxia.
Corrected

Reviewer: 2
Meaghan Ransom
Comments to the Author
Overall I felt this manuscript needed to be proofed for grammatical errors throughout before submitting. Minor examples like sometimes EV is written and other times it is spelled out, words missing between commas to merge sentences, etc. The abstract is not very clear and concise and I would suggest making substantial changes to the abstract. Specifically, the background sentences do not flow well. In the methods section it would help the reader to state these were a subset of patients. I would not include the comment about power in the conclusion section and discuss this as a limitation of the study later in the manuscript.
We agree and have now addressed each point. First, we performed a full proofread to correct the grammatical issues and enforce consistent terminology (e.g., consistent use of “EV” after first definition). Second, we rewrote the abstract to improve logical flow and precision, particularly in the background and conclusions. Third, we state in the Methods that the proteomics analysis was performed on a defined subset of the parent cohort, with the sampling/time window clearly specified. Finally, we removed the “insufficiently powered” statement from the abstract conclusion and instead state limitations in the Discussion (limitations paragraph).

Abstract now reads as follows: Background: “While cardiopulmonary transition in neonates is well described, the molecular changes which accompany it are not. The Extracellular Vesicles in Early preterm neonates and Thrombin generation (EVENT) study sought to explore this process molecular transition in preterm birth. How this differs from term born neonates is not well described.”

The main data figure is the highlight of the manuscript. Can you make the datapoints in B bigger and also the legend text bigger for B? For C, I would move the legend closer to the data.
Changes made for B, C figure is produced using a standardised package and legend cannot be brought significantly closer to heatmap. Apologies for this. We increased the datapoint size and label/legend font sizes in panel B. For panel C, legend size/position is constrained by the plotting function used to generate the heatmap; the submitted figure is a vector based graphic so is scalable to the size that is required by the journal.

Reviewer: 1
If you have selected ‘Yes’ above, please provide details of any competing interests.: Not applicable

Reviewer: 2
If you have selected ‘Yes’ above, please provide details of any competing interests.: Not applicable

VERSION 2 – REVIEW

**REVIEWER NAME**	Davenport, Patricia
**REVIEWER AFFILIATION**	

**REVIEWER CONFLICT OF INTEREST**	No Conflict of Interest
**AI DISCLOSURE**	No, I have not used AI
**DATE REVIEW RETURNED**	21-Apr-2026

**GENERAL COMMENTS**	The authors have greatly improved their manuscript with their edits. I have 2 very minor comments below.<br>1. While the introduction has been improved there are a few minor errors present (see below).<br>a. The following two sentences from the introduction do not make grammatical sense:<br>“The umbilical cord is cut reduces the total capillary bed and increasing systemic vascular resistance”<br>“This extends beyond the coagulation cascade, lymphocytes and platelets with differences also demonstrated between neonatal and adult monocytes and neutrophil function.”<br>b. The following sentence is repeated identically in two places in the introduction, please delete one: “Differences in coagulation profile has been noted in preterm infants compared to term infants in multiple studies, suggesting differences in proteomic content of blood.”

VERSION 2 – AUTHOR RESPONSE

Many thanks for your considered review.

We have corrected the submission as requested removing the duplicate sentence and clarifying the other sentences as follows:

“The umbilical cord is cut reduces the total capillary bed and increasing systemic vascular resistance” now reads as "The umbilical cord is then cut reducing the total capillary bed available for systemic blood flow and increasing systemic vascular resistance"

“This extends beyond the coagulation cascade, lymphocytes and platelets with differences also demonstrated between neonatal and adult monocytes and neutrophil function.” now reads "This molecular transition extends beyond the coagulation cascade, lymphocytes and platelets as described above with differences also demonstrated between neonatal and adult monocytes and neutrophil function"

We hope this increases the clarity of the manuscript as suggested
